# Using chi-squared automatic interaction detection modelling to identify student opinion profiles regarding same-sex couples as a family structure

**DOI:** 10.1016/j.heliyon.2021.e06469

**Published:** 2021-03-18

**Authors:** Clemente Rodríguez-Sabiote, José Álvarez-Rodríguez, Daniel Álvarez-Ferrandiz, Félix Zurita-Ortega

**Affiliations:** a Department of Research and Diagnosis Methods in Education, University of Granada, 18071 Granada, Spain; b Department of Pedagogy, University of Granada, 18071 Granada, Spain; c Department of Didactics of Musical, Plastic and Corporal Expression. University of Granada, Granada, Spain

**Keywords:** Family, Same-sex, Social sciences, Profiles, University students

## Abstract

The aim was to determine the opinions held by a sample of students in relation to homoparenting as a family modality. A cross-sectional study was conducted on a sample of university students specialization: social sciences using the AHFH. It is patent that opinions of students about the three factors (support, rejection and acceptance) that compose the construct of attitudes towards same-sex couples as a family structure, differ greatly depending on the positive or negative nature of these components. In conclusion, in relation to the dimension pertaining to rejection of same-sex couples as a family entity, we derived a configuration determined by 1 of the 4 predictor variables. In this case, gender was the only one of the 4 variables considered to support formation of a profile. This profile was constituted by male students who, independent of their birthplace setting, qualification and whether they personally know any same-sex couples, showed stronger agreement with the dimension describing rejection of same-sex couples as a family structure.

## 1 Introduction

It is evident that the concept of family as it is traditionally known has steadily changed, at least, as far as the context of the Western world is concerned. As a result, society must evolve in accordance with the context and era in which it exists, breaking with pre-existing prejudices and boundaries. In this regard, 2005 in Spain saw the introduction of the Law 13/2005 of the 1st of July which modified Civil Code in terms of one's rights to solicit marriage, alongside other rights such as joint paternity, inheritance and the right to a pension. This move supports a new type of family, a same-sex family (Ocón-Domingo et al., 2018; Gross, 2019). According to Gross (2003, cited by Bertocchi, 2017, p.275), the term is a “neologism that was created in the year 1997 in France by the Association of Gay and Lesbian Parents (APGL) in order to define family situations in which at least one parent self-identifies as homosexual”. In this sense, if we examine further we find that this word has been imported into Latin America without identifying the evolution of the concept, or the circumstances under which it emerged.

From this viewpoint, contributions from different authors were gathered from which a partial and exclusive nature is reflected in the construction of the term's definition, with some even indicating difficulties regarding social and family support (Belle et al., 2018; Kornatzki and Costa, 2019; Michaud and Stelmach, 2019; Hawthorne et al., 2020). In order to underline this statement, we refer to the definition provided by Ángulo et al. (2014, p.212), which states that: “Same-sex families are families within which the parental figures are made up by individuals of the same sex. They relate to gay and lesbian people who, as a couple, access maternity or paternity as families constituted by a gay or lesbian couple, or who educate and live with the children of one of the family's members”. This concept is based on a political conception, which seeks to make visible a set of fathers and mothers who were not previously included in the consideration of whether or not one is involved in parenting (Cohen, 2005).

Taking this Franco-Latin standpoint, Laguna (2016) indicates that in using this term we refer to the dichotomy between heteroparental and same-sex, giving the impression that one of the classifications (heteroparental) is considered to be superior to the other, given that it is linked to social reproduction. From this conception, the term could serve to present census data, without understanding the complex diversity that can be found amongst gay fathers and lesbian mothers (Farr et al., 2019; Ghosh, 2019; Watson et al., 2019).

The diverse way in which families are formed may be related to the choices made by citizens, with these varying as a function of their desires or circumstances. For this reason, one single model of the ideal family does not exist, with a considerable number of classifications and typologies instead being available (Powell et al., 2010; Gavriel-Fried and Shilo, 2017). In the present day, families can be seen with a wide array of typologies with regards to structure and organization, with each acting in different ways in their daily lives. With regards to structure and organisation, some family types can be indicated: extended families, single-parent families, reconstituted families, adoptive families, same-sex families, etc.

In consideration of the above and following the approval of homosexual marriage, a new type of family has recognized which we denominate as same-sex. These are families in which the progenitors are two people of the same sex who decide to be fathers or mothers. The existence of this type of family has generated controversy in Spain and other countries. On the one hand, this is due to the unfavourable position of homosexual couples and, on the other hand, to the traditional view of the family. Thus, the image of same-sex families represents a challenge to traditional models of parenting (Gavriel-Fried and Shilo, 2017; Robaldo, 2011).

The fundamental question posed is whether marriage between individuals of the same biological sex is essentially responsive to political-judicial issues and has a clear ideological component (Pawelski et al., 2006). In this sense, Ocón (2006) indicates that its acceptance [of marriage] created a large polemic determined by the ideologies, values and principles of its defenders and retractors, although it represents one of the most important legislative reforms of Spanish family law of recent times.

Currently, homosexual marriage has been legalised in 16 countries around the world. In others, such as Germany, Ireland, the Czech Republic, Austria, Luxembourg, Finland, Hungary and Slovakia, legality is manifested through common-law partnerships between individuals of the same sex. All in all, for decades the presence of homosexual groups has been seen to become more and more visible. In the present day, some laws exist that regulate the principle of equality which guarantees this EU European right. However, the reality is different in other countries, given that few detail rights of same-sex families in their legislative frameworks. In this sense, we allude to Latin America where a set of research studies have been conducted in relation to new family configurations, consisting of homosexual parenting (Robaldo, 2011), adaptive families (Salvo, 2016), studies about new parenting modalities (Lubeznov, 2018) and homoparenting (Muñoz, 2013; Viveros, 2017).

At a global level, this topic can be approached in the areas of law, education, domestic life, work life and perceptions. In this way, Bauer (2016) examined gender roles in domestic work in Austria, Belgium, France, Holland, Norway, Sweden and Australia. Kornatzki and Costa (2019) defined aspects of a judicial nature in Brazil, whilst Kridahl and Kolk (2018) have indicated aspects relating to retirement in Sweden, and work in Italy. This has fed into the redefinition of citizenship models proposed by Belle et al. (2018), and work models developed by Gavriel-Fried and Shilo (2017), which discuss social changes in the populations of Israel and the United States.

As pointed out in some studies related to this research topic, parenting styles can be a determining factor in the digital age. As evidenced in the researchs by Martínez et al. (2019) and Bi et al. (2018), there are several types of upbringing: *authoritarian* focus on obedience, punishment over discipline; *authoritative*, whose main purpose is to create positive relationship enforce rules; *permissive* focused on don't enforce rules (kids will be kids) and *uninvolved* that focuses your attention to provide little guidance nurturing, or attention. However, we show that regardless of the concept or family typology (nuclear, extensive, single parent, single parent, etc.) under consideration, we emphasize that from the point of view of future research, we must delve further into parenting styles and their influence on the behavior of children. In this regard, if we consider various studies from different contexts, for example, those of García et al. (2019) or Rosser-Limina, Suriá-Martínez, and Mateo-Pérez (2020); we can highlight the importance of parenting styles. Thus, the presence of more dialogue and authoritarian parents who employ strategies of acceptance, dialogue and participation with their children, decisively impact upon improvements in relation to different aspects of their lives. These can range from individual aspects to social, academic and psychological aspects, etc.

The aim of the present research sought to meet the following objectives: a) to determine the opinions held by a sample of students enrolled on different courses in relation to homoparenting as a family modality, and b) To establish student profiles which elucidate better or worse attitudes towards the aforementioned same-sex couples as a family type, breaking down these profiles according to latent factors comprising the measurement scale.

## 2 Methods

The research study developed was descriptive in nature. More specifically, it concerned a survey study which consisted of the administration of a Likert type scale. This scale measures attitudes towards same-sex couples as a family structure, in our particular case, the scale was applied to a representative sample of students enrolled on Education degrees at the University of Granada.

### 2.1 Research variables

The present research contemplated two types of variables. Firstly, we considered independent variables which were strictly attributive and non-experimental in nature. Amongst these, the following are found:

a) Gender: male vs female.
b) Qualification: Degrees of Social Education, Infant Education, Primary Education and Pedagogy.
c) Whether the respondent personally knows any same-sex couple/s, with whom they maintain interpersonal contact. We consider this contact as one that is established among the participants in our research with couples of the same sex in which relationships of empathy, reciprocity, understanding, etc.
d) Birthplace setting: rural vs urban.

Inclusion of these four variables is justified in the discussion and conclusion sections by the fact that these variables may have differential effects on the opinion of the students recruited to the present study. In the case of the variable ‘*knows any homoparental couple/s with whom they maintain interpersonal contact’*, we have highlighted classic theory regarding the contact hypothesis (Allport, 1954). Relevant empirical results are found in research studies conducted by Lemm (2006), Costa et al. (2015), Loehr et al. (2015), Collier et al. (2012) and Smith et al. (2009). These studies essentially posit that contact between members of different groups can help reduce prejudice and improve social relationships by promoting a more tolerant and integrated society.

Secondly, we considered the criterion variables or, in other words, each one of the 20 items that form the opinion scale relating to same-sex families as a family entity. However, these were grouped around a series of latent components or factors, from which inferences can be made following the preceptive exploratory factor analysis to which we will submit the obtained data, following scale administration.

### 2.2 Data collection

#### 2.2.1 Sample, sample characteristics and sampling process

The sample size was made up of 332 students of the Faculty of Education Sciences of University of Granada (Spain). It must be highlighted that stratified probabilistic sampling was used for sample selection, with qualification being taken as the stratum (Blair and Blair, 2015). Further, as has been indicated by Kalton (2020), the procedure used for determining sample size considered a series of basic aspects. In this sense, we must stress that we started from a reference population of approximately 6000 students, and used a confidence level of 0.95 and sampling error of ±5% with unknown probabilities p = q = 0.5. With regards to sample characteristics, mean age of participating students was 20.03 years old, with a standard deviation of 3.69, and corresponding to 19% males and 81%, females. With regards to birthplace setting, 39.2% were born in a rural setting and 60.8% in an urban environment. In addition, 37.7% did not personally know any same-sex families, whilst 62.3% reported that they did. Finally, with respect to the qualification being undertaken, 23.8% were enrolled on the degree of Social Education, 36.9% on a Primary Education degree, 26.8% on an Infant Education degree and the remaining 17.5% were studying for a Pedagogy degree. In the specific case of the degree of Pedagogy, at the University of Granada this course aims to train professionals in systems, institutions, contexts, resources, and educational and training processes. They also develop the personal, professional, social and cultural development processes that run alongside these in an integrated way in people and groups throughout life.

#### 2.2.2 Data collection instrument

An adaptation of the attitudes towards same-sex families scale [Actitud hacia las Familias Homoparentales (AHFH)] developed by Ramírez et al. (2006) was used for data collection. This scale consists of 20 items which are responded to along a Likert type scale with five possible response options. The scale runs from 1: Strongly disagree, to 5: Strongly agree. With regards to the number of alternative response options, Mata (2018) points to a line of work focused on analysing the way in which the number of response options affects the psychometric properties of Likert scales (Cox, 1980; Bishop, 1987; Dawes, 2008; Preston and Colman, 2000). This work concluded that reliability increases when the number of options were increased from five to seven. Nonetheless, this improvement is greater when increasing from four to seven options, with improvements being less notable above seven options (Dillman, 2007 and Finstad, 2010). For this reason we chose the 5-point scale introduced earlier, although we are aware that the provision of more response option could have increased reliability even further. On the other hand, we included 5 identification variables which acted as grouping variables in order to denote future differential effects. These variables are age, gender, qualification, birthplace setting and whether respondents personally know any same-sex families.

Further, the scale is composed of three dimensions which were indicated by prior exploratory factor analysis of the scale. The first dimension is acceptance of same-sex couples as a family structure and is formed by items 2, 10, 11, 13, 16, 17 and 19. The second dimension is rejection of same-sex couples as a family structure and is formed by items 1, 6, 7 and 9. Finally, support for same-sex couples as a family structure is formed by items 3, 4, 5, 8, 12, 14, 15, 18 and 20.

#### 2.2.3 Data collection procedure

A number of researchers were mobilised with the aim of administering the considered data collection scale. These researchers proceeded to administer the scale *in situ* in different classrooms where students were receiving classes on basic subjects. Teachers who were delivering these classes and their students provided consent for this strategy to be used. It followed that the various administrative duties and forms were completed at the beginning of each one of these aforementioned classes, within a time that did not exceed 15 min on any occasion. Administration of the scale lasted between two and three weeks. Evidently, the scale was totally anonymous and was voluntarily completed by the students who gave informed consent prior to participation. All collected information is held in a secure protected database and has been completely anonymised, consequently, guarantying all pertinent ethical and moral conditions.

### 2.3 Methodological rigour of the AHFH scale

Reliability and validity were considered in order to determine methodological rigour of the AHFH scale. In the first instance, reliability is contemplated as internal consistency given that the study involved only a single administration. For this we calculated the Cronbach α reliability coefficient, which achieved a value of α = 0.813. As a result, we can confirm that the AHFH scale is consistent and stable when administered to our sample, and that the different items that form it appear to convincingly measure the same latent construct. This is deducted because the items are highly intercorrelated (Revelle and Zinbarg, 2009; Sijtsma, 2009; Warrens, 2014).

With regards to the second parameter, validity is contemplated according to content validity, or the degree to which the items composing the scale really measure the concept for which they have been elaborated (Taherdoost, 2016). As a guarantee we took a previously elaborated scale that had already been used in a previous investigation (consult the previous section). We also contemplated concurrent criterion validity. For this, corrected item-total coefficients were calculated. This refers to the correlation between each individual item and a scale score (as an internal criteria) that excludes that item. In all cases, correlation coefficients of r > 0.35 were achieved. According to some scientists (Cook and Beckman, 2006; Heale and Twycross, 2015; Campos et al., 2017; Solans-Domènech et al., 2019), this is sufficient empirical evidence to be able to confirm that each item individually contributes to the scale (univocity). Finally, exploratory factor analysis was performed with a double objective. To a certain extent, it endows the scale with better construct validity and, to another extent, it infers the latent factors within which the 20 items forming the scale are organised, with these providing the dependent variables. The main results from the conducted factor analysis are shown in the following sections of the paper (extraction method, adequacy of the Pearson correlations matrix and explained variance and interpretation of the exploratory factor analysis).

#### 2.3.1 Extraction method

Principal component with Kaiser criterion (λ ≥ 1), rotation applied: Varimax. With regards to the statistical results obtained prior to implementation of exploratory factor analysis, we highlight the determinant of the correlation matrix of |*A*| = .0008935. In obtaining a value close to 0, but not a null value, we can guarantee that the resulting correlation matrix is not a singular matrix and that the linear equations associated to the matrix could have a solution.

#### 2.3.2 Adequacy of the Pearson correlations matrix

Moreover, the value for the Kaiser-Meyer-Olkin measure of overall sampling adequacy was KMO = .829. According to Pituch and Stevens (2016) and Holmes-Finch (2019), this can be considered as an acceptable value which enables us to proceed with the exploratory factor analysis. Individual measures of sampling adequacy also obtained values of MSA≥.75 in all cases, with all being greater than the minimum value of measure of sampling adequacy, MSA = .5 (Kline, 2014; Tabachnick and Fidell, 2018). These results are a valid indicator that that bivariate correlations for all items are larger than the values obtained for the partial correlations. In addition, the value produced from the Bartlett test of sphericity obtained a value of χ^2^ = 1842.535(df = 190; p = 0.000). This means that the correlation matrix obtained is not an identity matrix, or in other words, it is not a matrix in which correlations are perfect along the diagonal (intercorrelated items, r = 1) and null elsewhere in the matrix.

#### 2.3.3 Explained variance and interpretation of the exploratory factor analysis

In relation to results of the exploratory factor analysis implemented, we highlight the presence of 3 factors which, when taken together, explain a variance (explained σ^2^) of 43.32%. The commonalities obtained for each one of the scale variables are all located at intervals of h^2^ = .25-.75. This denotes that all of them together and each one individually is well represented in the resulting factor solution (Pituch and Stevens, 2016; Flake et al., 2017), although it is the case that some variables are better represented than others. With regards to the resulting factor solution, we must point out that factor loadings reached saturation with an r>±.35 (at least 10% explained variance, practical significance criterion).

In reference to the components or factors obtained, we highlight a primary factor which is composed of 9 items (explained σ^2^ = 25.58%) and whose common aspect concerns items formulated in favour of same-sex couples as a family structure. This factor could be denominated as ‘Support’ for same-sex couples as a family state.

We also identified a secondary factor constituted by 4 items (explained σ^2^ = 9.86%) and whose common aspect speaks to disagreement with same-sex couples as a family unit. As such, we could denominate this factor as ‘Rejection’ of same-sex couples as a family state.

Finally, we also found a tertiary factor which is curiously composed of more items than factor 2, specifically 7 items. Its explained σ^2^, however, was 7.88%, with this being lower than for factor 2. The items that form this factor are related with acceptance of same-sex couples as a family structure, as much in the context of friendships, as in school, social and general settings. In this way, we could denominate factor 3 as ‘Acceptance’ of same-sex couples as a family modality.

Finally, in order to avoid potential bias that could affect validation of the prediction models inferred previously via the CHAID (Chi-Squared Automatic Interaction Detection) method, invariance analysis was carried out through confirmatory analysis, specifically, adjusted maximum likelihood (ML) estimations. This analysis was conducted according to gender and carried out using the program jamovi v.1.2 (The jamovi project, Sydney, Australia). Gender differentiated outcomes were as follows (see Table 1):

**Table 1:** Fit measures of confirmatory factor analysis (CFA) females vs. males.

Gender	CFI	TLI	RMSEA	RMSEA 90%CI	
				Lower	Upper
Females	0.899	0.918	0.0787	0.0664	0.091
Males	0.789	0.897	0.0667	0.0566	0.0773

Note: CFI: comparative fit index, TLI: Tucker–Lewis index, RMSEA: root mean square error of approximation.

Both confirmatory factor analyses produced empirical evidence of the presence of a 3-factor structure. Outcomes were similar between males and females, with highly similar fit values also being produced. Firstly, incremental fit indices pertaining to the comparative fit index (CFI) values were 0.899 and 0.789 for females and males, respectively. Whilst non-normed fix indices or Tucker–Lewis indices (TLI) were 0.918 and 0.897 for females and males, respectively. According to Schermelleh-Engel et al. (2003), these values can be considered to reasonably indicate good fit. Secondly, the absolute fit measure of the root mean square error of approximation (RMSEA) obtained higher values for both genders than the 0.06 cut-point advised by Hu and Bentler (1999). Nonetheless, the root mean square error of approximation (RMSEA) value has a known statistical distribution that can also be calculated via confidence intervals (lower vs upper), from confidence intervals of 90% and above. In light of this, if the lower limit of the confidence interval is higher than 0.05 and the upper limit is lower than 0.09, as was the case in the present study, we can consider fit to be reasonably good (Browne and Cudeck, 1993). In conclusion, we can conclude that our prediction models, developed using the CHAID (Chi-Squared Automatic Interaction Detection) method, demonstrate full validity as shown by empirical evidence supporting the lack of gender-based variance.

## 3 Data analysis and interpretation

The quantitative data analysis program SPSS v.26 (IBM, Armonk, NY, USA), was used to analyse information collected by the previously described scale. Inverse items were recoded as part of a preliminary procedure prior to further analysis and, in addition, four *dummy* variables were created to serve as dependent variables when creating the three contemplated profiles. These variables are:

a) Latent factor 1→ Support for same-sex couples as a family structure formed by the sum of the mean values of the items that compose it, these being: 3, 4, 5, 8, 12, 14, 15, 18 and 20.
b) Latent factor 2→ Rejection of same-sex couples as a family structure formed by the sum of the mean values of relevant items, these being: 1, 6, 7 and 9.
c) Latent factor 3→ Acceptance of same-sex couples as a family structure formed by the sum of the mean values of relevant items, these being: 2, 10, 11, 13, 16, 17 and 19.

To achieve the objective a): To determine the opinions held by a sample of students enrolled on different courses in relation to homoparenting as a family modality.

It can be appreciated that the factor pertaining to support for same-sex couples as a family structure received broad support, with levels seen to oscillate between a minimum mean value of 4.41 and a maximum value of 4.76, with an overall mean of 4.61. Given that these scores were produced from a scale with a direct maximum score of 5, this finding is fairly noteworthy. Moreover, the dimension pertaining to acceptance of same-sex couples as a family unit also obtained moderate agreement, although the overall mean value obtained was almost a point lower (mean = 3.73) than the support factor, and the minimum and maximum mean values were 3.52 and 3.81, respectively. Finally, the factor pertaining to rejection of same-sex couples as a family structure received, without doubt, least agreement with an overall mean of 1.68, minimum mean of 1.56 and maximum mean of 2. Notwithstanding the aforementioned, some differences are seen between the numerical means constituting the diverse levels of the four variables, which will act as predictors when developing profiles. For this reason, it was considered relevant to develop a multivariate technique capable of configuring profiles which reflect the characteristics of each one of the contemplated factors.

To ensure objective b) To establish student profiles which elucidate better or worse attitudes towards the aforementioned same-sex couples as a family type, breaking down these profiles according to latent factors comprising the measurement scale.

From these precedents we implemented a type of multivariate analysis suitable for the development of CHAID (Chi-Squared Automatic Interaction Detection) profiles. Use of multiple linear regression that uses three latent variables as a criterion (in three separate models) and identification variables as predictors is interesting. The implementation of multiple correspondence analysis would also have been possible and the results obtained through the CHAID (Chi-Squared Automatic Interaction Detection) technique would, most likely, be essentially coincident. However, the CHAID (Chi-Squared Automatic Interaction Detection) technique offers at least five advantages in relation to multiple linear regression (Lizasoain et al., 2003). Firstly, it is capable of handling both quantitative and qualitative variables. This is useful as it is not advisable to use non-metric predictors in multiple linear regression analysis. Secondly, the geographical results of CHAID (Chi-Squared Automatic Interaction Detection), decision trees, are very simple to interpret for profiling, this being the fundamental purpose of our research. Thirdly, the CHAID (Chi-Squared Automatic Interaction Detection) technique facilitates the identification of interactions. This is because predictor variables are used in relation to each other and also allows the characterization of subpopulations. Fourthly, the tables and graphs used to present results are easily interpreted which is very important for the presentation and communication of results to non-expert audiences. Lastly, in contrast to multiple linear regression, it is not necessary when using the CHAID (Chi-Squared Automatic Interaction Detection) technique to fulfill assumptions of homocedasticity, normality, multicollinearity and independence. In summary, it is a useful exploratory tool that allows users to come up with guidelines for designing more refined models for subsequent analysis.

Realisation of this technique requires a categorical or ordinal dependent variable (our four cases) and various independent variables or categorical predictors (in our case, four predictors: qualification, gender, birthplace setting and whether or not the respondents personally knew any same-sex couples) which, combined, enable identification of segments or divisions from which profiles can be elaborated (Onoja et al., 2018; Van Diepen and Franses, 2006). We can, therefore, state that it is a technical statistic which analyses the interaction between a criterion variable and multiple non-metric predictor variables, and uses the Bonferroni χ^2^ statistic as a dividing criterion (Byeon, 2017; Munandar and Winarko, 2015). The main disaggregated results according to the three profile models contemplated following application of this technique, are presented next.

### 3.1 Model 1: profiles associated with the dependent variable: support same-sex couple by independent variables gender, birth environmental, knowledge same-sex couple and degree

**Figure 1:**
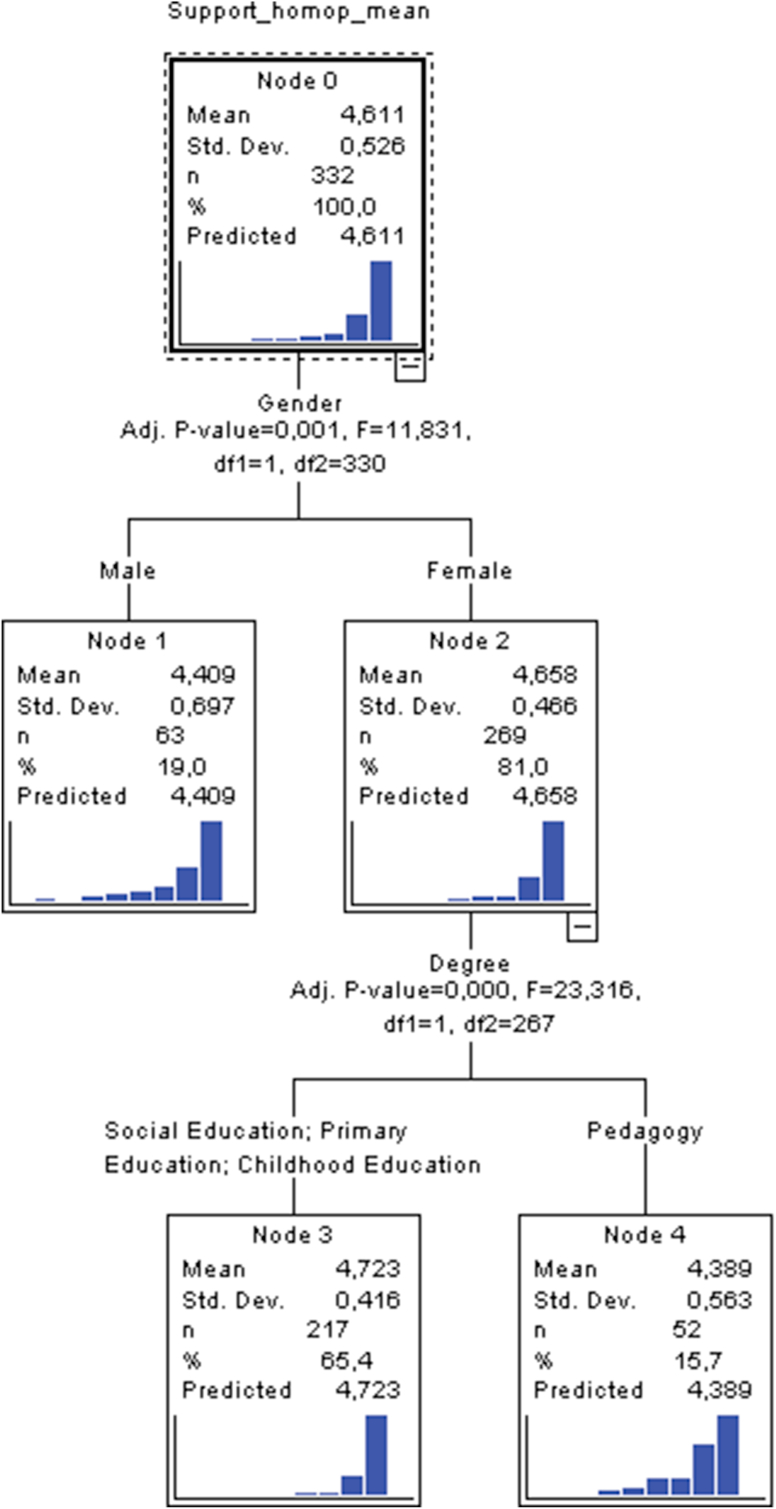
Tree plot of model 1.

As shown in Table 2, Table 3 and Figure 1, enables evaluation of outcomes when the criterion variable describing support of same-sex couples as a family structure is crossed with the four considered predictors. It can be seen that only two of them acquire the role of variables permitting configuration of a characteristic profile: Gender and qualification, with a total of 5 nodes, 3 terminal nodes and a depth of 2 nodes. Thus, from model 1 we can infer up to a total of 5 nodes, if we consider the initial node (node 0) as irrelevant to the effects of profile elaboration. In nodes 1 and 2, the first variable that permits a first characteristic profile to be illustrated is the gender variable. This first segmentation differentiates two nodes, the first node (node 1) being associated with the male gender, relative to node 2 that refers to females. Significance testing (Bonferroni χ ^2^) comparing means reported by female participants (mean = 4.65) with means reported by male participants (mean = 4.40) produced an empirical value of F = 11.831 (1.330; p_adj_. = .000). Thus, in this first division sufficient empirical evidence exists to consider the female gender as being the dominant influence at this level and demonstrating greatest support towards same-sex couples as a family structure.

**Table 2:** Averages obtained for each latent factor disaggregated by gender, degree, scope of birth and whether or not you know a same-sex couple.

**Gender**	**Support homop.**	**Acceptance homop.**	**Against homop**.∗
Male	4.40	3.58	2
Female	4.65	3.76	1.60
Total	4.61	3.73	1.68
**Birth environmental**	**Support homop.**	**Acceptance homop.**	**Against homop.**∗
Rural Environment	4.65	3.74	1.65
Urban Environment	4.58	3.72	1.70
Total	4.61	3.73	1.68
**Do you know a same-sex couple?**	**Support homop.**	**Acceptance homop.**	**Against homop.**∗
No	4.52	3.63	1.74
Yes	4.66	3.79	1.64
Total	4.61	3.73	1.68
**Degree**	**Support homop.**	**Acceptance homop.**	**Against homop.**∗
Social Education	4.76	3.81	1.56
Primary Education	4.64	3.78	1.75
Childhood Education	4.56	3.72	1.72
Pedagogy	4.41	3.52	1.63
Total	4.61	3.73	1.68

∗ The averages have been calculated without reversing the values of the reverse items.

**Table 3:** Model 1 summary.

Specifications	
Growing Method	CHAID
Dependent Variable	Support_homop_mean
Independent Variables	Gender, Birth_environmental, Knowlegde_Same-sex_Couple, Degree
Validation	None
Maximum Tree Depth	3
Minimum Cases in Parent Node	100
Minimum Cases in Child Node	50
Results	

Independent Variables Included	Gender, Degree
Number of Nodes	5
Number of Terminal Nodes	3
Depth	2

If we inquire further, we can appreciate the presence of a second segmentation which is split into two nodes. The first of these is node 3 which is associated with all considered specialties (mean = 4.72) and the second is node 4 which is related with the degree course of Pedagogy (mean = 4.38). Comparison of means obtained a Bonferroni χ^2^ value of F = 23.16 (1.267; p_adj_. = .000). This produces sufficient empirical evidence to consider that female students enrolled on the specialties of Social Education, Primary Education and Infant Education demonstrate stronger agreement with same-sex couples as a family entity than students undertaking a Pedagogy degree, who showed much lower support (see Tables 4,5,6,7,8).

**Table 4:** Tree Table of model 1.

Node	Mean	Std. Deviation	N	Percent	Predicted Mean	Parent Node	Primary Independent Variable					
							Variable	Sig.^a^	F	df1	df2	Split Values
0	4.61	.52	332	10%	4.61							
1	4.40	.69	63	19%	4.40	0	Gender	.001∗∗∗	11.831	1	330	Male
2	4.65	.46	269	81%	4.65	0	Gender	.001∗∗∗	11.831	1	330	Female
3	4.72	.41	217	65.4%	4.72	2	Degree	.000∗∗∗	23.316	1	267	Social Education; Primary Education; Childhood Education
4	4.38	.56	52	15.7%	4.38	2	Degree	.000	23.316	1	267	Pedagogy

Growing Method: CHAID (Chi-Squared Automatic Interaction Detection).

Dependent Variable: Acceptance_homop_mean

*∗p < .05∗∗p < .01∗∗∗p < .001*.

a Bonferroni adjusted.

**Table 5:** Model 2 summary.

Specifications	
Growing Method	CHAID
Dependent Variable	Acceptance_homop_mean
Independent Variables	Gender, Birth_environmental, Knowlegde_Same-sex_Couple, Degree
Validation	None
Maximum Tree Depth	3
Minimum Cases in Parent Node	100
Minimum Cases in Child Node	50
Results	

Independent Variables Included	Knowlegde_Same-sex_Couple
Number of Nodes	3
Number of Terminal Nodes	2
Depth	1

**Table 6:** Tree Table of model 2.

Node	Mean	Std. Deviation	N	Percent	Predicted Mean	Parent Node	Primary Independent Variable					
							Variable	Sig.^a^	F	df1	df2	Split Values
0	3.73	.56	332	100%	3.73							
1	3.63	.54	125	37.7%	3.63	0	Knowlegde_Same-sex_Couple	.012∗	6.31	1	330	No
2	3.79	.57	207	62.3%	3.79	0	Knowlegde_Same-sex_Couple	.012∗	6.31	1	330	Yes

Growing Method: CHAID (Chi-Squared Automatic Interaction Detection).

Dependent Variable: Acceptance_homop_mean

*∗p < .05∗∗p < .01∗∗∗p < .001*.

a Bonferroni adjusted.

**Table 7:** Model 3 summary.

Specifications	
Growing Method	CHAID
Dependent Variable	Against_homop
Independent Variables	Gender, Birth_environmental, Knowlegde_Same-sex Couple, Degree
Validation	None
Maximum Tree Depth	3
Minimum Cases in Parent Node	100
Minimum Cases in Child Node	50
Results	

Independent Variables Included	Birth environmental
Number of Nodes	3
Number of Terminal Nodes	2
Depth	1

**Table 8:** Tree Table of model 3.

Node	Mean	Std. Deviation	N	Percent	Predicted Mean	Parent Node	Primary Independent Variable					
							Variable	Sig.^a^	F	df1	df2	Split Values
0	1.68	.673	332	100%	1.68							
1	2.00	.931	63	19%	2.00	0	Gender	.000∗∗∗	18.419	1	330	Male
2	1.60	.572	269	81%	1.60	0	Gender	.000∗∗∗	18.419	1	330	Female

Growing Method: CHAID (Chi-Squared Automatic Interaction Detection).

Dependent Variable: Acceptance_homop_mean

*∗p < .05∗∗p < .01∗∗∗p < .001*.

a Bonferroni adjusted.

### 3.2 Model 2: profiles associated with the dependent variable: acceptance same--sex couple by independent variables gender, birth environmental, knowledge same-sex couple and degree

**Figure 2:**
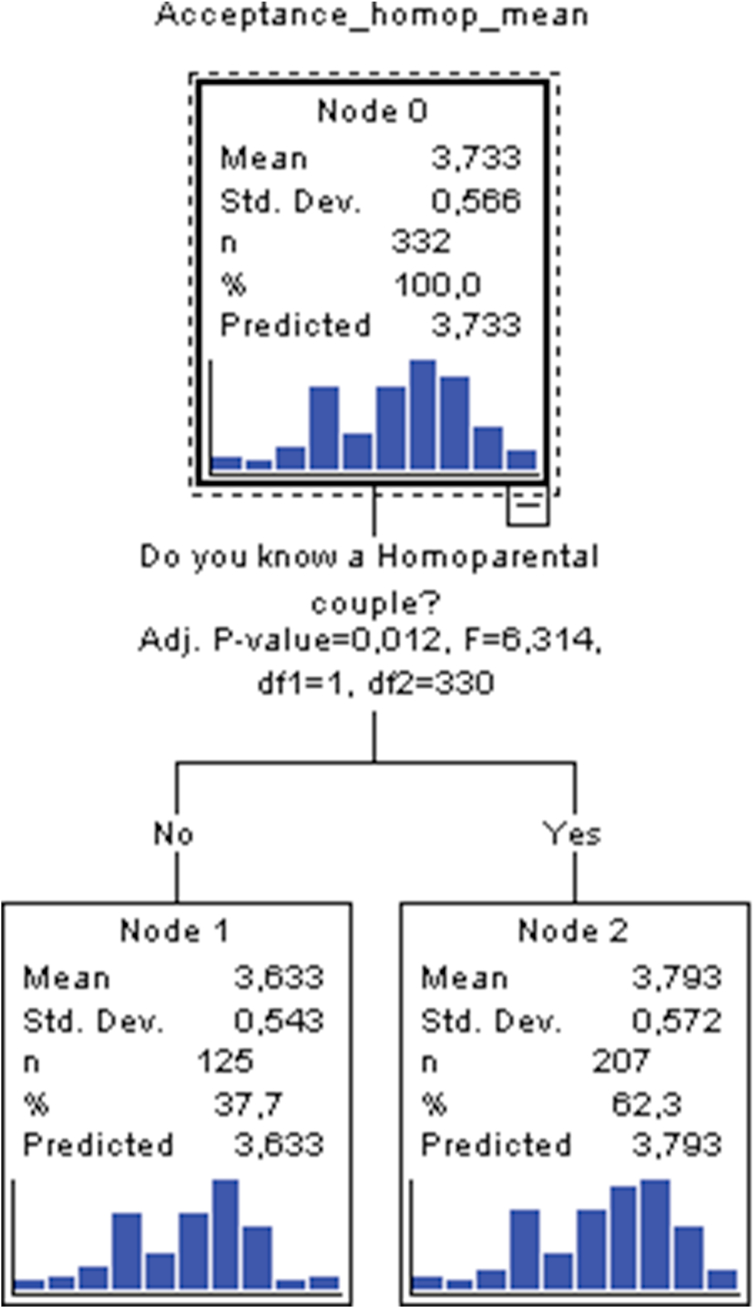
Tree plot of model 2.

With regards to results obtained for the factor describing acceptance of same-sex couples as a family structure it can be appreciated that, of the four contemplated predictors, only one of them acquires the role of a contributing variable to elaboration of a characteristic profile. This is the predictor pertaining to whether one personally knows any same-sex couple. It has a total of 3 nodes, with 2 terminal nodes and a depth of 1. Up to a total of 3 nodes can be inferred from model 2, depending on whether we consider the initial node (node 0) to be inconsequential to the effects of profile elaboration. In nodes 1 and 2, the first and only variable that permits delineation of a first characteristic profile is the variable pertaining to personally knowing a same-sex couple. Two nodes are derived in this first and only segmentation. The first is formed by node 1 which is associated with those students who do not personally know a specific same-sex couple, relative to node 2 which is tied to students who, in contrast, do know a same-sex couple. Significance testing (Bonferroni χ^2^) comparing means of those who do not know this type of couple (mean = 3.63) with the means of those who do (mean = 3.79), uncovered an empirical value of F = 6.314 (1.330; p_adj_. = .012). From this first and only division, therefore, sufficient empirical evidence exists to consider that students who personally know a same-sex couple, regardless of gender, qualification or birthplace setting, are more accepting of same-sex couples as a family type than students who do not know any such couples (see Figure 2).

### 3.3 Model 3: profiles associated with the dependent variable: against same-sex couple by independent variables gender, birth environmental, knowledge same-sex couple and degree

**Figure 3:**
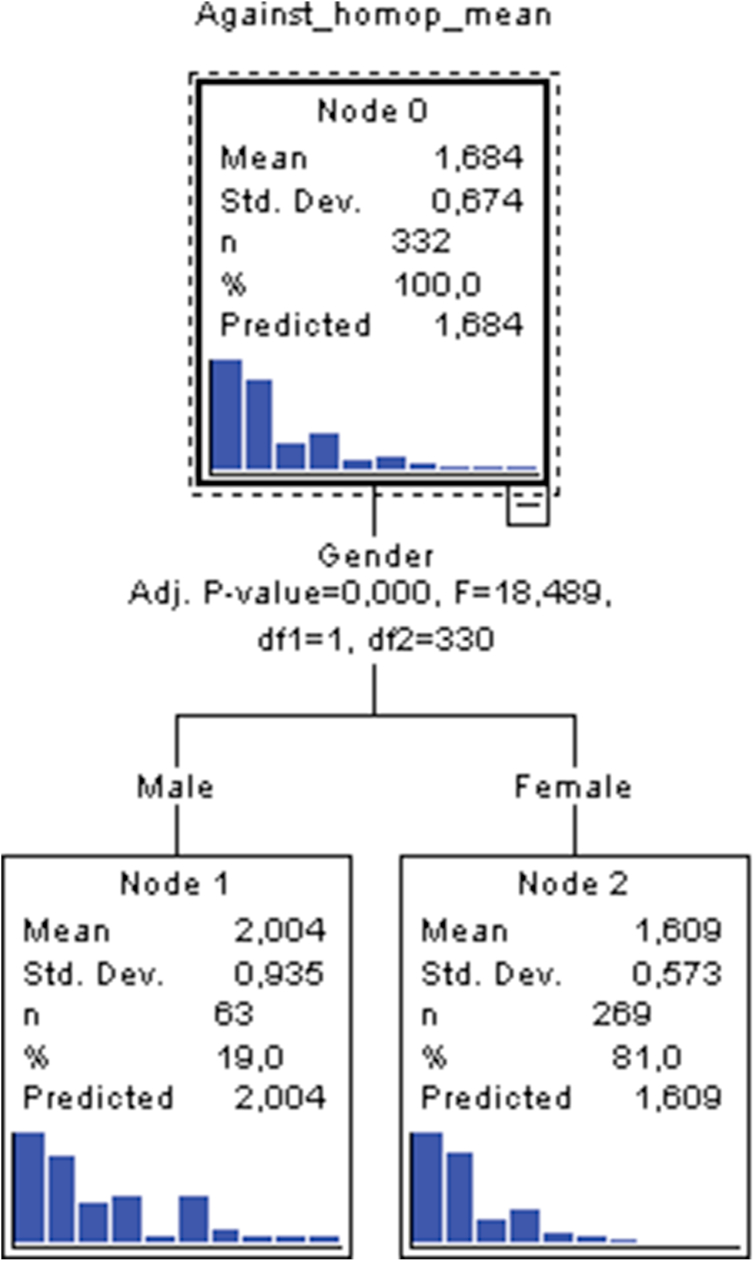
Tree plot of model 3.

In reference to results obtained for the factor pertaining to rejection of same-sex couples as a family unit, four factors can be contemplated though only one of these assumes the role of a contributing variable in construction of a characteristic profile. This useful factor is the gender of students, with a total of 3 nodes, 2 terminal nodes and a depth of 1, as was the case with the acceptance dimension. In model 3, up to a total of 3 nodes are inferred, not counting node 0, whose contribution to the effects of profile elaboration is null. For nodes 1 and 2, the first and only variable that permits a first characteristic profile to be traced is the gender of participating students. This first and only segmentation is split into two nodes. The first is formed by node 1 which is associated with male students, relative to node 2 which is linked to female students. Significance testing (Bonferroni χ^2^) comparing means reported by male participants (mean = 2) with means reported by females (mean = 1.60) obtained an empirical value of F = 18.419 (1.330; p_adj_. = .000). In this first and only branch, sufficient empirical evidence exists to consider that male students show stronger agreement than female students with responses of rejection towards same-sex couples as a family structure. This was the case independent of whether or not they personally knew any same-sex couple, their qualification or birthplace setting (see Figure 3).

## 4 Discussion and limits of study

If we frequently use this important term of homoparenting, we run the risk of forgetting the valuable and enrichening practices that take place within parental relationships between sexually and, resultantly, emotionally diverse individuals. Amongst these individuals we can cite trans, bisexual and intersexual individuals, potentially creating a discourse of invisibility towards them (Laguna, 2016).

In recent decades, Spain has experienced relevant changes at both a social and cultural level, which have contributed to the evolution of the family structure. For this very reason, as society changes, it is necessary for mindsets to also change and for society to welcome new times. In this way, diversity and inclusion will be achieved for all types of families that emerge, such as reconstituted families, single parent families and same-sex families, amongst others. Some authors indicate the importance of educating families from an early age in order to positively impact upon the personalities of adolescents (Moreno et al., 2009).

Current ease in soliciting divorce following acceptance and approval of the Divorce Law 15/2005, of the 8th of July, which modified Civil Code and the Civil Procedural Law regarding separation and divorce, means that many marriages access this law. Many of these couples go on to find other individuals with whom they restart their lives. In this way, reconstructed families are formed, changing the view of single mothers and creating single parent families. We have mentioned that at the root of the approval of homosexual marriage and the law allowing the possibility of adopting children, same-sex families have emerged. All of these changes have led to a new image and concept of family (Loaisa et al., 2019).

With regards to limits of the study the study developed presents some limitations, above all, those that are methodological in nature. In this sense, we must highlight a series of future perspectives whose inclusion could lead to improvements. Firstly, we highlight the local context of the implemented research which was limited to a single university centre. With respect to this fact, we believe it would be appropriate to increase the sample size of the research topic, diversifying and broadening the qualifications and sampling points to other geographical zones.

The sample was also seen to be highly gender unbalanced with females being over-represented relative to males. However, this is explained by the fact that the faculty under investigation contains a large majority of females. In this sense, it is an essential task for future research studies to achieve greater balance in study samples males and females.

We are convinced that this aspect would, undoubtedly, legitimize the study conclusions derived, bestowing upon them a greater power of generalization. Moreover, it is also considered necessary to consider other identification variables which are predictive in nature, in addition to those considered here. These will help us to form opinion profiles regarding same-sex couples as more complete and inclusive family structures.

Regardless of the concept or typology of the family (nuclear, extensive, single-parent, same-sex, etc.), which we consider, we emphasize that from the point of view of future research, we must deepen the parental styles and their influence on the behavior of children. In this regard, if we take into account some studies, from different contexts, for example (Martínez et al., 2019; Rosser-Limiñana et al., 2020; Salvador-Pérez et al., 2019) we can highlight the importance of parenting styles. Thus, the presence of more dialogue and authorized parents who offer more strategies of acceptance, dialogue and participation with their children decisively influences the improvement of different aspects of their lives ranging from the individual, social, academic, psychological, etc.,

Finally, another aspect that could help improve the study is the implementation of other multivariate analysis procedures which are correlational-predictive and causal in nature, such as structural equation models. The objective of this would be to determine which variables play a preponderant role in determining a better or worse predisposition of university students towards same-sex couples as family units.

## 5 Conclusions

With respect to results obtained in the present study, and responding to the research objectives raised, it is patent that opinions of students about the three factors (Support, Rejection, Acceptance) that compose the construct of attitudes towards same-sex couples as a family structure, differ greatly depending on the positive or negative nature of these components. Thus, when factors with positive connotations are considered, specifically, ‘Support’ and ‘Acceptance’ factors towards same-sex couples as a family unit, reported means indicate agreement scores that fall between high (in the case of support) and medium (in the case of acceptance). In contrast, when the factor possesses negative connotations (rejection of same-sex families as a family entity), disagreement is much more pervasive. This consideration may be due to a number of causes, amongst the most important within young people attending university is the presence of progressive ideologies, alongside their lack of prejudice in relation to this and other social issues (Massey et al., 2015; Ioverno et al., 2018).

Nonetheless, given that which has just been explained and bearing in mind the four predictor variables considered for the development of profiles, we derived certain differences between the levels that make up these variables. This provides sufficient justification to encourage further research into this topic. In this way, following application of the CHAID (Chi-Squared Automatic Interaction Detection) technique we were able to derive that various differential characteristic profiles essentially exist, which consider each one of the contemplated factors.

In this regard, firstly and in relation to the dimension of ‘Support’ of same-sex couples as a family structure, we discovered a profile composed of 2 of the 4 predictor variables (gender, degree, interpersonal contact with the same-sex couple and context). Students who most strongly agreed with items showing support to this family variation were shown to be female and enrolled on the courses of Social, Infant and Primary Education. This result is similar to that produced by various previously developed studies (Ocón-Domingo et al., 2018; Webb and Kavanagh, 2016; Baiocco et al., 2013; Vecho, Gross, Gratton, D'Amore and Green, 2019).

Secondly, in regards to what is referred to as the dimension of ‘Acceptance’ of same-sex couples as a family unit, we find a group of students characterised by only 1 of the 4 predictor variables. In this way, students who showed stronger agreement with ‘Acceptance’ were composed of students who, regardless of gender, birthplace setting and qualification, fundamentally have in common interpersonal contact with same-sex couples who make up a family unit. This finding is congruent with that which is denominated the contact hypothesis. It refers to those individuals who possess direct interpersonal contact with same-sex couples and evidence greater acceptance towards them. An abundant number of students corroborated this hypothesis (Costa et al., 2015; Lemm, 2006; Loehr et al., 2015; Collier et al., 2012; Smith et al., 2009).

In conclusion, in relation to the dimension pertaining to rejection of same-sex couples as a family entity, we derived a configuration determined by 1 of the 4 predictor variables. In this case, gender was the only one of the 4 variables considered to support formation of a profile. This profile was constituted by male students who, independent of their birthplace setting, qualification and whether they personally know any same-sex couples, showed stronger agreement with the dimension describing rejection of same-sex couples as a family structure. This was in contrast to female students, who were less likely to report consistently with this factor.

## Declarations

### Author contribution statement

Clemente Rodríguez-Sabiote: Conceived and designed the experiments; Performed the experiments; Analyzed and interpreted the data; Wrote the paper.

José Álvarez-Rodríguez, Daniel Álvarez-Ferrándiz and Félix Zurita-Ortega: Contributed reagents, materials, analysis tools or data; Wrote the paper.

### Funding statement

This research did not receive any specific grant from funding agencies in the public, commercial, or not-for-profit sectors.

### Data availability statement

Data included in article/supplementary material/referenced in article.

### Declaration of interests statement

The authors declare no conflict of interest.

### Additional information

No additional information is available for this paper.

## References

[bib1] Allport G.W. (1954). The Nature of Prejudice.

[bib2] Ángulo A., Granados J., González M. (2014). Homoparental families experiences with psychology professionals in Mexico, Distrito Federal. A qualitative approximation. Cuicuilico.

[bib3] Baiocco R., Nardelli N., Pezzuti L., Lingiardi V. (2013). Attitudes of Italian heterosexual older adults towards lesbian and gay parenting. Sex. Res. Soc. Pol..

[bib4] Bauer G. (2016). Gender roles, comparative advantages and the life course: the division of domestic labor in same-sex and different-sex couples. Eur. J. Popul..

[bib5] Belle E., Peroni C., Rapetti E. (2018). One step up and two steps back? The Italian debate on secularization, heteronormativity and LGBTQ citizenship. Soc. Compass.

[bib6] Bertocchi F. (2017). The state of studies and research on the homosexual parent family in Italy. Ital. Sociol. Rev..

[bib7] Bi X., Yang Y., Li H., Wang M., Zhang W., Deater-Deckard K. (2018). Parenting styles and parent-adolescent relationships: the mediating roles of behavioral autonomy and parental authority. Front. Psychol..

[bib8] Bishop G.F. (1987). Experiments with the middle response alternative in survey questions. Publ. Opin. Q..

[bib9] Blair E., Blair J. (2015). Applied Survey Sampling.

[bib10] Browne M.W., Cudeck R., Bollen K.A., Long J.S. (1993). Alternative ways of assessing model fit. Testing Structural Equation Models.

[bib11] Byeon H. (2017). Chi-square automatic interaction detection modeling for predicting depression in multicultural female students. Int. J. Adv. Comput. Sci. Appl..

[bib12] Campos C.M., Da Silva Oliveira D., Feitoza A.H., Cattuzzo M.T. (2017). Reliability and content validity of the organized physical activity questionnaire for adolescents. Educ. Res..

[bib13] Cohen C., En Johnson P., Herdenson M.G. (2005). Punks, bulldaggers, and welfacew queen: the radical potential od Queer politics. Black Queer Studies. EUA.

[bib14] Collier K.L., Bos H.M., Sandfort T.G. (2012). Intergroup contact, attitudes toward homosexuality, and the role of acceptance of gender non-conformity in young adolescents. J. Adolesc..

[bib15] Cook D.A., Beckman T.J. (2006). Current concepts in validity and reliability for psychometric instruments: theory and application. Am. J. Med..

[bib16] Costa P., Pereira H., Leal I. (2015). The contact hypothesis and attitudes toward same-sex parenting. Sex. Res. Soc. Pol..

[bib17] Cox E. (1980). The optimal number of response alternatives in a scale: a review. J. Mark. Res..

[bib18] Dawes J. (2008). Do data characteristics change according to the number of scale points used? An experiment using 5 point, 7 point and 10 point scales. Int. J. Mark. Res..

[bib76] Dillman D.A. (2007). Mail and internet surveys: The tailored design method.

[bib19] Farr R.H., Bruun S.T., Simon K.A. (2019). Family conflict observations and outcomes among adopted school-age children with lesbian, gay, and heterosexual parents. J. Fam. Psychol..

[bib20] Finstad K. (2010). Reponse interpolation and scale sensitivity: evidence against 5-point scales. J. Usability Stud..

[bib21] Flake J.K., Pek J., Hehman E. (2017). Construct validation in social and personality research: current practice and recommendations. Soc.. Psychol. Personal. Sci..

[bib22] García F., Serra E., García O.F., Martinez I., Cruise E.A. (2019). Third emerging stage for the current digital society? Optimal parenting styles in Spain, the United States, Germany, and Brazil. Int. J. Environ. Res. Publ. Health.

[bib23] Gavriel-Fried B., Shilo G. (2017). The perception of family in Israel and the United States: similarities and differences. J. Fam. Issues.

[bib24] Ghosh A. (2019). After coming out: parental acceptance of young lesbian and gay people. Soc. Compass.

[bib25] Gross M. (2003). L’Homoparentalité.

[bib26] Gross M. (2019). Métamorphoses de la parente. París.

[bib27] Hawthorne O., Camic P.M., Rimes K.A. (2020). Understanding the structure, experiences and challenges of social support for older lesbian, gay and bisexual people: a systematic review. Ageing Soc..

[bib28] Heale R., Twycross A. (2015). Validity and reliability in quantitative studies. Evid. Base Nurs..

[bib29] Holmes Finch W. (2019). Exploratory Factor Analysis.

[bib30] Hu L., Bentler P. (1999). Cut-off criteria for fit indexes in covariance structure analysis: conventional criteria versus new alternatives. Struct. Equ. Model..

[bib31] IBM Corp. Released (2019). IBM SPSS Statistics for Windows, Version 26.0.

[bib32] Ioverno S., Baiocco R., Lingiardi V., Verrastro V., D’Amore S., Green R.J. (2018). Attitudes towards same-sex parenting in Italy: the influence of traditional gender ideology. Cult. Health Sex..

[bib33] Kalton G. (2020). Introduction to Survey Sampling.

[bib34] Kline P. (2014). An Easy Guide to Factor Analysis (E-Book).

[bib35] Kornatzki L., Costa P.R. (2019). The formation of the family in contemporary Brazil: an analysis of laws and legal decisions. Rev. Ibero-Am Estudos Educ..

[bib36] Kridahl L., Kolk M. (2018). Retirement coordination in opposite-sex and same-sex married couples: evidence from Swedish registers. Adv. Life Course Res..

[bib37] Laguna O.E. (2016). Review of homoparental concepts and homoparental family: achieves and limits from a view of relatioships and parental links of the people of the sexual diversity. Rev. Estud. Género Ventana.

[bib38] Lemm K.M. (2006). Positive associations among interpersonal contact, motivation, and implicit and explicit attitudes toward gay men. J. Homosex..

[bib39] Lizasoain L., Joaristi L., Santiago C., Lukas J.F., Moyano N., Sedano M., Munárriz B. (2003). Use and tecniques if segmentation in the evaluation of the performance in languages. A study of the Comunidad Autónoma Vasca. Rev. Invest. Educ..

[bib40] Loaisa F., Rodríguez C., Álvarez D., López J.A., Álvarez J. (2019). Initial study about the perception to homoparental families in the province of Granada, Spain. Rev. Espacios.

[bib41] Loehr A., Doan L., Miller L.R. (2015). The role of selection effects in the contact hypothesis: results from a U.S. National survey on sexual prejudice. Arch. Sex. Behav..

[bib42] Lubeznov E., Nitzan K., Stolowitz D., Levy A., Boruchovitz-Zamir R., Diamond G. (2018). Measuring adult children’s perceptions of their parents’ acceptance and rejection of their sexual orientation: initial development of the parental acceptance and rejection of sexual orientation scale (PARSOS). J. Homosex..

[bib43] Martínez I., Murgui S., García O.F., García F. (2019). Parenting in the digital era: protective and risk parenting styles for traditional bullying and cyberbullying victimization. Comput. Hum. Behav..

[bib44] Massey S., Merriwether A., García J. (2015). Modern prejudice and same-sex parenting: shifting judgments in positive and negative parenting situations. J. GLBT Fam. Stud..

[bib45] Matas A. (2018). Likert-type scale format design: state of art. Rev. Electrón. Invest. Educ..

[bib46] Michaud C.K., Stelmach B. (2019). Lesbian and gay parents' experiences and their relationships with/in schools: an alberta study. Can. J. Educ. Adm. Pol..

[bib47] Moreno D., Estévez E., Murgui S., Musitu G. (2009). Relation between the family climate and scholar climate: empathy role, attitude to the authority and the violent behavior in the adolescence. Int. J. Psychol. Psychol. Ther..

[bib48] Munandar T.A., Winarko E. (2015). Regional development classification model using decision tree approach. Int. J. Comput. Appl..

[bib49] Muñoz F. (2013). The main nucleus of the society: the arguments againts the homoparental upbringing in the cases Atala and Peralta. Rev. Ius et Praxix.

[bib50] Ocón J. (2006). Adoptive family and changes in the traditional family organization. Pap..

[bib51] Ocón-Domingo J., Rodríguez-Sabiote C., Álvarez-Ferrándiz D. (2018). Opinion profiles of the university students about the homoparentality in nowadays-familiar metamorphosis. OBETS. Rev. CSS.

[bib52] Onoja A.A., Babasola O.L., Ojiambo V. (2018). Chi-square automatic interaction detection modeling of the effects of social media networks on students' academic performance. J. Bus. Manag..

[bib53] Pawelski J.G., Perrin E.C., Foy J.M., Allen C.E., Crawford J.E., Del Monte M. (2006). The effects of marriage, civil union, and domestic partnership laws on the health and well-being of children. An. Pediatr..

[bib54] Pituch K.A., Stevens J.P. (2016). Applied multivariate statistics for the social sciences. Analises with SAS and IBM´s SPSS.

[bib55] Powell B., Bolzendahl C., Geist C., Steelman L.C. (2010). Counted Out: Same-Sex Relations and Americans’ Definitions of Family.

[bib56] Preston C.C., Colman A. (2000). Optimal number of response categories in rating scales: reliability, validity, discriminating power, and respondent preferences. Acta Psychol..

[bib57] Ramírez S., Moliner V., Vicent L. (2006). Attitudes towards homoparental families in the scholar context. Fòrum Recerca.

[bib58] Revelle W., Zinbarg R.E. (2009). Coefficients alpha, beta, omega, and the GLB: comments on Sijtsma. Psychometrika.

[bib59] Robaldo M. (2011). The homoparentality in the deconstruction and reconstruction of the family. Notes for a discussion. Rev. Punto Gen..

[bib60] Rosser-Limiñana A., Suriá-Martínez R., Mateo Pérez M.Á. (2020). Children exposed to intimate partner violence: association among battered mothers’ parenting competences and children’s behavior. Int. J. Environ. Res. Publ. Health.

[bib61] Salvador-Pérez F., Muros-Molina J.J., Gámiz-Sánchez V., Zurita F. (2019). Systematic review of the relationship of healthy life habits in childhood and adolescence and its influence on health. ESHPA – Educat. Sport, Health Phys. Activ..

[bib62] Salvo I. (2016). Construction of maternity in adoptions single parents: mandates, wishes and elections. Rev. Psicol..

[bib63] Schermelleh-Engel K., Moosbrugger H., Müller H. (2003). Evaluating the fit of structural equation models: tests of significance and descriptive goodness-of-fit measures. Methods Psychol. Res. Online.

[bib64] Sijtsma K. (2009). On the use, the misuse, and the very limited usefulness of cronbach’s alpha. Psychometrika.

[bib65] Smith S., Axelton A., Saucier D. (2009). The effects of contact on sexual prejudice: a meta-analysis. Sex. Roles.

[bib66] Solans-Domènech M., Pons J.M.V., Adam P., Grau J., Aymerich M. (2019). Development and validation of a questionnaire to measure research impact. Res. Evaluat..

[bib67] Tabachnick B.G., Fidell L.S. (2018). Using Multivariate Statistics.

[bib68] Taherdoost H. (2016). Validity and reliability of the research instrument; how to test the validation of a questionnaire/survey in a research. Int. J. Acad. Res. Manag..

[bib69] The JAMOVI Project (2020). JAMOVI (version 1.2) [computer software]. https://www.jamovi.org.2020.

[bib70] Van Diepen M., Franses P.H. (2006). Evaluating chi-squared automatic interaction detection. Inf. Syst..

[bib71] Vecho O., Gross M., Gratton E., D´Amore S., Green R.J. (2019). Attitudes toward same-sex marriage and parenting, ideologies, and social contacts: the mediation role of sexual prejudice moderated by gender. Sex. Res. Soc. Pol..

[bib72] Viveros E.F. (2017). Apology for diversity. About the case of the marriage of homosexual couples in Colombia. Rev. Univ. Católica Luis Amigó.

[bib73] Warrens M.J. (2014). On cronbach’s alpha as the mean of all possible k-split alphas. Advan. Stat..

[bib74] Watson R.J., Rose H.A., Doull M., Adjei J., Saewyc E. (2019). Worsening perceptions of family connectedness and parent support for lesbian, gay, and bisexual adolescents. J. Child Fam. Stud..

[bib75] Webb S., Kavanagh P. (2016). Attitudes toward same-sex parenting: an effect of gender. J. Homosex..

